# Effectiveness and Estimation of Cost-Effectiveness of a Group-Based Multicomponent Physical Exercise Programme on Risk of Falling and Frailty in Community-Dwelling Older Adults

**DOI:** 10.3390/ijerph16122086

**Published:** 2019-06-13

**Authors:** Tamara Alhambra-Borrás, Estrella Durá-Ferrandis, Maite Ferrando-García

**Affiliations:** 1 Polibienestar Research Institute, Universitat de València, 46022 Valencia, Spain; estrella.dura@uv.es; 2 Kveloce I+D+i, R&D+i Consultancy, 46002 Valencia, Spain; mferrando@kveloce.com

**Keywords:** risk of falling, frailty, falls, older adults, physical exercise, ageing

## Abstract

This study analyses the effectiveness and cost-effectiveness of a group-based multicomponent physical exercise programme aimed at reducing the risk of falling and frailty in community-dwelling older adults. This is a pretest–posttest non-equivalent control group design, with an intervention group and a comparison group. Participants were evaluated at baseline and after 9 months. The effectiveness analyses showed significant reduction in the risk of falling (−45.5%; *p* = 0.000) and frailty (−31%; *p* = 0.000) after the intervention for the participants in the physical exercise programme. Moreover, these participants showed an improvement in limitations in activities of daily living, self-care ability and the use of health resources, physical performance, balance and body mass index. The cost-effectiveness analyses showed that the intervention was cost-saving and more effective than usual care scenario. A novel group-based multicomponent physical exercise programme showed to be more effective and cost-effective than usual care for older adults suffering from risk of falling and frailty.

## 1. Introduction

Falls and frailty are crucial health issues affecting older adults. Both share many significant domains and represent a major burden on the healthcare system.

Falls represent an important and complex health problem because of its high prevalence among older adults and its consequences at physical, psychological and social level [1]. One in three people over 65 falls at least once a year worldwide [2]. According to the European Injury Data Base [3], among older adults 58% of emergency departments attendance in hospitals are due to falls; 2.3 million people over 65 are estimated to be treated every year in an emergency department because of a fall, whereof approximately 1.5 million are admitted to the hospital. The health care expenditure for treating fall-related injuries in the EU is estimated to be 25 billion euros each year.

On the other hand, frailty is the most problematic expression of population ageing. Traditionally, frailty has been defined as a medical syndrome consisting of solely physical components—frailty phenotype framework [4]. However, in the last decade, a multidimensional integral perspective of frailty has been introduced defining frailty as “a dynamic state affecting an individual who experiences losses in one or more domains of human functioning (physical, psychological, social) that are caused by the influence of a range of variables and which increases the risk of adverse outcomes.” [5]. Moreover, frailty can be seen as a relative state that may change over time [6,7]. Although frailty is a complex condition which increases with age, no consensus exists about the prevalence rates of frailty. This is partly due to the operational definition of frailty used. Among those studies that used the physical phenotype, prevalence varied from 4% to 17%; while in the studies that measured frailty using comprehensive definitions, frailty prevalence ranged from 4.2% to 59.1%.

Both falls and frailty have a proven association with mortality, morbidity, hospitalisations, reduced mobility, limitations in activities of daily living (ADLs), disability, fear of falling, a reduced self-care ability, as well as social isolation and a reduced quality of life [8,9,10,11,12]. Furthermore, falls and frailty and their consequences have proven to increase health care utilisation and expenditures [13,14].

The high prevalence and the consequences on health of the risk of falling and frailty among older people highlight the need to develop and to implement strategies that effectively address these conditions. In this regard, the regular practise of physical activity has proved to prevent and reduce frailty and the risk of falling, even the injuries resulting from falls [15,16]. Balance and strength training have showed to be the most effective kinds of training [17,18]. In this regard, the OTAGO programme [19] has been identified as one of the most effective multicomponent programmes [20,21].

In this study, the purpose was to analyse the effect of a group-based multicomponent physical exercise programme on the reduction of the risk of falling and frailty in community-dwelling older adults. This programme offers a modified format of OTAGO programme including evidence-based improvements. On the one hand, 26 additional exercises to the 34 existing ones are included in the multicomponent programme. The additional exercises were designed by a physiotherapist and included dynamic self-resistance exercises. These kinds of exercises are designed to harmoniously develop the muscles of the body without using any equipment but using the body as a resistance. Muscle strength, as well as balance, play a central role in fall and frailty prevention and their consequences [17,18]. On the other hand, as part of OTAGO, a walking plan of two 30-minute walks per week is included. However, our programme does not include walking prescription because several systematic reviews of the literature have pointed out that programmes that do not include walking plans as part of their intervention are more effective on risk of falling than those including walking plans [17,18]. Moreover, our programme, instead of being individually prescribed and delivered at participant’s home, offers a group-based exercise programme, which has previously shown to motivate participants to perform exercises more effectively [22,23]. Finally, the vast majority of studies using the OTAGO programme have been focused on reducing falls and related injuries, and our study also explores as a primary outcome the impact on frailty. 

Furthermore, this study analysed the impact of the intervention on other health-related variables, namely limitations in ADLs, health-related quality of life, self-care agency, self-efficacy related to falls and the use of health resources; being the hypothesis that after intervention, participants would also show an improvement in these variables. In addition, a secondary objective was to estimate the cost-effectiveness of the intervention in order to know if our physical exercise programme was potentially cost-saving in comparison to usual care for older people who were frail or at risk of falling.

## 2. Materials and Methods

### 2.1. Study Design

This prospective longitudinal study followed a quasi-experimental design, concretely a pretest–posttest non-equivalent control group design, with an intervention group and a comparison group, where participants were not randomly assigned to the two groups. Participants included in this study were evaluated at baseline and after 9 months.

All participants included in the study provided written informed consent. The design and informed consent procedure of the study were approved by the Ethics Committee of the Consorcio Hospital General Universitario de Valencia (Spain, 20131201).

### 2.2. Study Participants and Inclusion Criteria

The study population consisted of community-dwelling people aged 65 years or older from the city of Valencia (Spain) with no severe physical or cognitive limitations. These two exclusion criteria were assessed by general practitioners from both healthcare centres participating in the study.

From February 2015 to July 2015 a total of 500 participants from two healthcare centres in the city of Valencia were invited to participate in the study. Each of these healthcare centres corresponded at convenience to one of the study groups: intervention (*N* = 258) and comparison group (*N* = 242).

Participants were screened for risk of falling and/or frailty by individual assessments carried out at each participant’s home by previous appointment and after accepting to participate in the study at the healthcare centre. No statistically significant differences were found in any of the relevant demographic and health-related variables between the intervention group and the comparison group. After this baseline assessment those participants from both healthcare centres meeting the inclusion criteria (having risk of falling and/or frailty) were included in the study. Among participants from healthcare centre 2 (where the intervention was conducted), acceptance to participate in the physical exercise programme was also established as an inclusion criterion. Therefore, subjects from healthcare centre 2 had to agree to enrol into the intervention prior to be included as part of the intervention group, being this one of the main reasons why the intervention group sample was reduced from 258 to 74.

**Figure 1:**
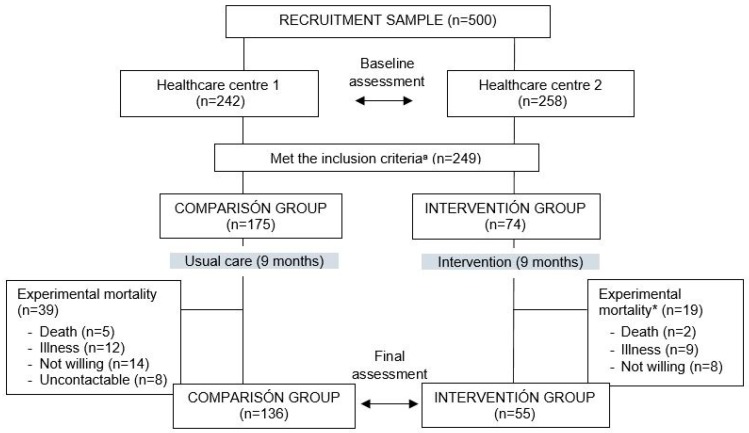
Flow of participants through the study. ^a^ Inclusion criteria: Having risk of falling and/or frailty and, for intervention group, acceptance to participate in the physical exercise programme. * Experimental mortality includes the participants who dropped out of the study after having met the inclusion criteria.

A total of 175 participants composed the comparison group and 74 participants composed the intervention group (Figure 1). 

### 2.3. Outcome Measures

#### 2.3.1. Primary Outcomes

Risk of falling was assessed among participants using the following criteria recommended by the American Geriatrics Society & British Geriatrics Society [24]: (1) had fallen at least twice during the previous 12 months; (2) had self-reported walking problems; (3) had self-reported balance problems (criteria 2 and 3 were assessed using questions 3 and 4 of the TFI [6]). Moreover, a fourth criterion on having a fear of falling [25] was also included, as suggested by several studies [26,27]. Participants were classified as having risk of falling if they answered yes on criterion 1 or yes on criterion 4 plus yes on one or both criteria 2 and 3. The responses obtained from these questions were dichotomised to yes or no for risk of falling.

Frailty was assessed through the Tilburg Frailty Index (TFI) [6]. The TFI’s total score ranges from 0 to 15. The higher the score, the higher one’s frailty. Frailty is diagnosed with a TFI score ≥5. The TFI is based on a multidimensional approach to frailty, including physical, psychological and social aspects, and it showed a robust evidence of reliability and validity [28]. Cronbach’s alpha was 0.69 for the participants in this study.

#### 2.3.2. Secondary Outcomes

Limitations in activities of daily living were measured using the Groningen Activity Restriction Scale (GARS) [29]. Cronbach’s alpha for the study participants was 0.91. Self-care agency, as the individual’s ability to evaluate health-related needs and conduct self-care activities aimed at promoting and maintaining health and well-being [30], was evaluated using the Spanish version of the Appraisal of Self-care Agency Scale-Revised (ASA-R) [31] and Cronbach’s alpha was 0.77 for the respondents. Besides, participants’ self-efficacy related to falls was measured through the Falls Efficacy Scale-International (FES-I) [32], which Cronbach’s alpha was 0.83 for the study participants. This questionnaire is intended to assess fear of falling among older people. Fear of falling may cause the reduction of activities, leading to a reduced physical activity and mobility, which paradoxically increase the risk of falls and injury [33,34]. Participants’ quality of life was measured using the SF-12 Health Survey [35]. SF-12 Cronbach’s alpha for the study participants was 0.90. The use of healthcare resources was also evaluated at baseline and after 9 months, asking participants an estimation of all doctor visits and hospitalisations over the previous 12 months (at baseline) or the previous 9 months (at post-intervention).

Furthermore, the intervention group participants—those included in the physical exercise programme—were also assessed through two extra measures: physical performance and body composition. Physical performance was evaluated using the Short Physical Performance Battery (SPPB) [36] which includes the balance test, the gait speed test and the chair stand test. Finally, body composition, including weight, body mass index (BMI), body fat (kg), lean body mass (kg) and body water (kg), was measured using a Tanita scale (TBF300), a measure of bioelectrical impedance.

Finally, for the cost-effectiveness analyses of the intervention the following costs were estimated: the cost related to the physical exercise programme (costs of the physiotherapist in charge of the programme implementation and supervision) and the healthcare costs related to the use of healthcare resources (number of doctor’s visits and number of hospitalisations in both intervention and comparison groups). Furthermore, the quality of life, which was measured using the SF-12 Health Survey, was also used for these analyses.

### 2.4. Intervention

Participants allocated in the intervention group enrolled in a 9 months physical exercise programme. During this period, participants met twice a week in sessions of 45 min. In each session an exercise routine was performed and supervised by a physiotherapist. All intervention sessions were conducted in a community centre located in the participants neighbourhood.

The physical exercise programme was a multicomponent intervention including both balance and strength training, which have showed to be the most effective kind of training in preventing falls and frailty [2,18,26,37,38]. A total of 60 exercises conformed the programme, from which 34 exercises were based on the OTAGO programme [19], one of the most successful programmes for preventing falls [21], and the other 26 exercises were designed ad hoc by a physiotherapist. The ad hoc exercises include dynamic self-resistance training, which is a specific strength training that involves flexing the muscles hard while also moving. The programme comprises a total of ten routines with 6 exercises each one. It is important to highlight that the OTAGO programme was originally designed to be implemented individually at home, while we adapted it to be delivered in group sessions. Physical exercise in a group format has proven to be as effective, or even more effective, than individual sessions [22,23]. A detailed description of the physical exercise programme implemented in this study has been included as Supplementary Materials S1.

### 2.5. Data Analyses

Before the effectiveness analyses, we conducted analyses on the experimental mortality in order to know if people who dropped out differ significantly from participants that stayed in. These analyses were conducted for the intervention variables (risk of falling and frailty) using contrast test Chi-square tests for the risk of falling and t-test for frailty.

The effectiveness analysis of the intervention was conducted through the analyses of the risk of falling and frailty. Intragroup analyses were performed to know the evolution of these variables in the comparison and the intervention group, and intergroup analyses to compare the two groups in two different moments: pre-intervention and 9 months later.

Given the categorical nature of the risk of falling, intergroup analyses were performed using Chi-square test, and intragroup differences were analysed using McNemar test. As frailty was a continuous variable a Repeated Measures ANOVA test was selected to analyse intragroup and intergroup differences. However, this analysis showed that the variable frailty did not meet the homoscedasticity criterion. Thus a transformation of the variable using Box-Cox power transformation was carried out. However, after an exponential transformation, the frailty variable still did not meet the homoscedasticity criterion. So, non-parametric test was selected. In particular, Wilcoxon’s signed rank test was used to calculate intragroup differences and Mann–Whitney U test for intergroup differences.

The impact of the intervention on other health-related variables—physical performance, body composition, limitations in ADLs, self-care agency, self-efficacy related to falls, quality of life and use of healthcare resources—was also measured using McNemar test and the Wilcoxon test, as these variables did not meet the assumptions of the parametric test.

Finally, the potential cost-effectiveness of the intervention was estimated by means of the Monitoring and Assessment Framework for the European Innovation Partnership on Active and Healthy Ageing (MAFEIP) tool [39]. Concretely, incremental costs and effects analyses, and cost-effectiveness analyses were carried out using this framework, which provides a web application enabling cost-effectiveness estimations based on the principles of Decision Analytic Modelling (DAM) and the traditional Markov model to assess the impact of innovations on health outcomes and resource use [39]. To provide the input required by MAFEIP, the intervention and usual care costs, and the utility (quality of life weight) were estimated. The central element in the cost-estimation were the human resources expenses (physiotherapist) required for the programme implementation, which was calculated to be 1560 euros (1.5 h/week × 52 weeks × 20 euros/h) for an average of 15 participants attending the programme sessions, which results in an intervention cost per person of 104€. On the other hand, the costs related to the use of healthcare resources (frequency of doctor’s visits and hospitalisation) during the study (9 months) was also estimated for both intervention and comparison groups. In order to calculate the costs of doctors’ visits, an average rate of 29.23€ per hour was assumed (based on the national statistics on social security for this professional category). The costs estimation per hospital bed-day was 733.56€ (calculated from the data on the hospital inpatient curative care expenditure and the number of hospital curative care bed-days; both variables available in Eurostat for Spain). With these assumptions, health resources consumption costs were estimated per group at two states, baseline and deteriorated, using MAFEIP. Healthcare costs for patients in baseline state were estimated to be 1615.02€ for the intervention group and 1630.22€ for the comparison group; while for those in deteriorated state were 3130.96€ for intervention group and 9030.13€ for the comparison group. To calculate the effects on health-related quality of life, the EQ-5D is recommended to be used within the MAFEIP context. However, this study did not use this questionnaire, instead the SF-12 Health Survey was used [35]. Thus, SF-12 scores were converted into EQ-5D scores using an algorithm developed by Gray, Rivero-Arias and Clarke [40].

## 3. Results

The age of the participants ranged from 65 to 92 years old and it included both females (73.2%) and males (26.8%). Intervention group participants were slightly younger than comparison group participants (mean age 76.7 vs. 78.4) and the percentage of women was higher in the intervention group (81.8% vs. 64.7%). Among those who dropped out (experimental mortality), no statistically significant differences were found in risk of falling and frailty neither for the intervention group (risk of falling *p* = 0.85; frailty *p* = 0.08) nor for the comparison group (risk of falling *p* = 0.63; frailty *p* = 0.55). The exercise group mean attendance throughout the study was 85% of the planned sessions. A session was considered successfully finished when all exercises were completed.

After attending the physical exercise programme intervention group participants presented a significant reduction in both the risk of falling and frailty. The risk of falling was reduced by 45.5% (*p* = 0.000) among the older people who attended the intervention. On the other hand, in the same time period, comparison group participants suffered a slight increase in falls risk, but it was not statistically significant (*p* = 1.000). Moreover, intergroup differences were significant before and after the intervention. In fact, the intervention participants showed higher prevalence of risk of falling at baseline, while after the intervention period the comparison group presented higher scores on falls risk (Table 1). In the case of frailty, similar results as for the risk of falling were found. The intervention demonstrated a significant reduction of frailty of 31% (*p* = 0.000) among intervention group participants, while the comparison group did not show a statistically significant reduction in frailty (*p* = 1.000). Furthermore, intergroup differences also showed a reverse trend before and after intervention in both groups (Table 2).

Physical performance and body composition were assessed only among intervention group participants (Table 3). Participants in the physical exercise programme showed an improvement in all the variables measured through the Short Physical Performance Battery. This improvement was found statistically significant for the overall scale (*p* = 0.009), which measures physical performance, and for the balance test (*p* = 0.009). The impact of the physical exercise programme on the body composition was moderated, even though all the body-related variables were slightly improved. This improvement was only found statistically significant for the body mass index (BMI), which showed a reduction of 0.4 points after intervention (*p* = 0.45).

The impact of the intervention on the health-related variables measured in this study was moderated (Table 4). Intervention group participants improved slightly but not significantly the quality of life (SF-12 Physical Health *p* = 0.200; SF-12 Mental Health *p* = 0.535) compared to the comparison group who presented a significant worsening of the quality of life after 9 months (SF-12 Physical Health *p* = 0.001; SF-12 Mental Health *p* = 0.003). Both groups presented a better ability for self-caring after the intervention period (intervention group *p* = 0.001; comparison group *p* = 0.000). The intervention group also presented a significant improvement in the limitation in ADLs, which were reduced by 6% (*p* = 0.011) after the intervention, while the comparison group experienced an increase of these limitations over the same period, but it was not statistically significant (*p* = 0.905). The average number of doctor visits was significantly reduced among intervention group participants (*p* = 0.000). Prior to be included in the intervention, this group visited the doctor an average of 8.8 times per year, while after the intervention this average was reduced to 5.9 visits per year. Hospitalisation was also reduced by 50% (*p* = 0.508) among intervention group participants and increased by 14% (*p* = 0.832) in the comparison group, but these numbers were not found statistically significant.

Finally, cost-effectiveness analyses of the intervention were conducted using the MAFEIP tool. The previous results on the intervention effect were introduced together with the costs assumptions in the web-based tool to provide utility estimations for the two different health status as recommended by the framework; this is baseline health status and deteriorated health status [39]. Table 5 shows the cost and utility values used in the model.

**Figure 2:**
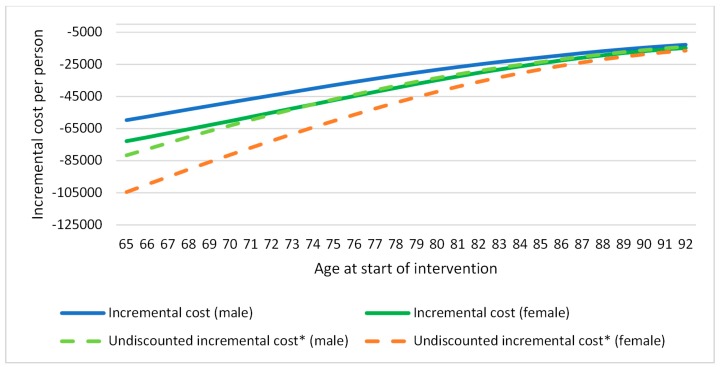
Incremental cost by age (healthcare). Figure 2 presents incremental costs for every age-gender combination in the specified target population. * Undiscounted incremental costs are those not applying the discount factors for costs and effects which are used to estimate outcomes while taking into account the future costs and health effects. This means adjusting for differences in the timing of costs (estimated expenditure) compared to health benefits (outcomes). The discount factor rate applied to this study was 3.5% for both cost and outcomes.

**Figure 3:**
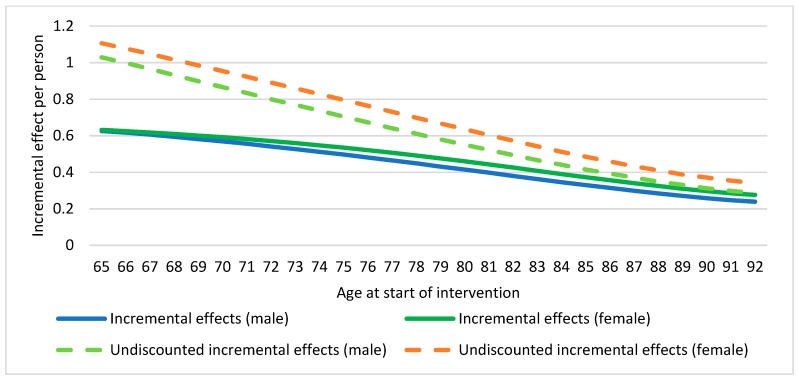
Incremental effects by age. Figure 3 presents incremental effects for every age-gender combination in the specified target population.

Incremental costs analysis referred to the difference between the cost that a person from a specific age and gender would have if he/she received the intervention minus the cost that would have if he/she followed usual care. The results of this analysis showed that incremental costs by age were negative (Figure 2) meaning that, for older adults suffering from risk of falling and/or frailty, usual care was more expensive than the intervention. Moreover, the upward trend of the graphic meant that the older the participant the less savings. On the other hand, the incremental effects values referred to how much quality of life (utility) was gained when the intervention was used instead of the usual care. These were also showed per age–gender group. The results of the incremental effects by age were also positive, due to the fact that the physical exercise programme increased quality of life and health outcomes among participants (Figure 3). In this case, the graphic showed a downward trend meaning that the improvement was less noticeable as the age increased.

**Figure 4:**
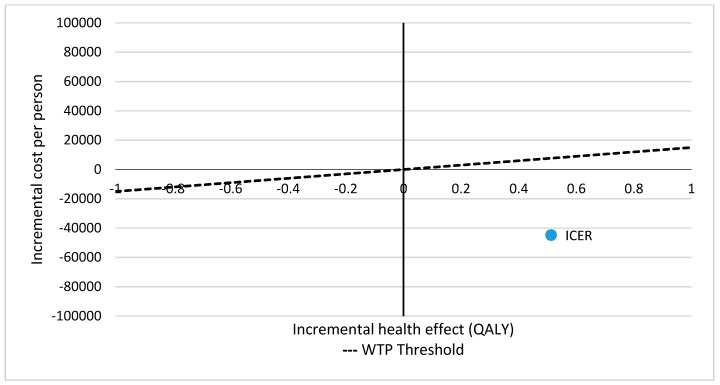
Cost-effectiveness plane (healthcare costs). Figure 4 presents the overall impact of the intervention on the costs and effects of the whole target population ICER: incremental cost-effectiveness ratio; WTP Threshold: willingness to pay (WTP) threshold per quality-adjusted life-year (QALY).

The incremental cost-effectiveness ratio (ICER) represented the overall impact of the physical exercise programme on costs and quality-adjusted life years (QALYs) for the total target population. The cost-effectiveness results are shown in both Table 6 and Figure 4. In this case, the ICER was in the lower-right quadrant (Figure 4). This position of the ICER indicated that the physical exercise programme was cost-effective in comparison to the usual care alternative. The diagonal line represents the willingness to pay (WTP) per additional QALY gained, which is the maximum amount that a patient is willing to give in exchange for a better quality of life.

## 4. Discussion

The effectiveness analyses results showed that our intervention significantly reduced the risk of falling by 45.4% after attending for 9 months the physical exercise programme; while in the same time period the comparison group suffered a slight increase in the risk of falling (+0.8%). This reduction is higher than the one showed in other studies. The studies where the OTAGO Programme was validated reported a reduction of falls up to 35% and the systematic review of 159 studies carried out by Gillespie et al. [2] indicated that interventions based on multicomponent exercise programmes reduced the risk of falling about 30%.

In regard to frailty, our physical exercise programme showed a statistically significant reduction of frailty of 31% among intervention group participants, while the comparison group experienced a reduction of 4%, which has not been found statistically significant. In the absence of consensus on the operational definition of frailty, the different studies that have tried to understand the effects of an exercise programme on frailty assessed frailty based on different criteria [4]. Therefore, the comparison of our results on frailty with the ones found in other studies presents certain difficulties.

Regarding the secondary outcomes, our work showed significant improvement in physical performance, in line with systematic reviews [41,42,43,44]. In most of these studies, physical performance was measured by SPPB, the one used in our study. After our intervention, intervention group participants presented improvements in all SPBB three tests, being this improvement only significant for the balance. In contrast, the systematic revision carried out by Giné-Garriga et al. [43] concluded that exercise programmes applied to frail older adults significantly improved the gait speed and chair stands test, while this improvement did not occur in the balance test. In addition, the revision of Cadore et al. [37] also found improvements in gait speed and in balance and Haider et al. [44] found improvements in balance. Perhaps the absence of significant improvement in gait speed among the intervention group participants in this study was due to the fact that our physical exercise programme was mainly focused on balance training and strength training.

As for body composition, which has also been used as a criterion of frailty in some research, our study showed a significant reduction in BMI in the participants of the physical exercise programme. Among the studies that have included body composition as a measure of frailty, the review of Theou et al. [45] found no significant results for this criterion in frail older people after participating in an intervention based on exercise.

Limitations in ADLs have been also included in many of these studies as a criterion of frailty since it has been found as both a risk factor and a consequence of falls [46] and frailty [10,47]. Accordingly, and as expected, after the intervention, the intervention group presented a significant reduction of ADLs limitations. Similar results were found in the systematic revisions of Chou et al. [48] and Theou et al. [45].

In regard to the other health variables related to falls and frailty, the results confirmed that our intervention had effects beyond the intervention variables: the intervention group experienced greater improvements in self-care agency, in self-efficacy related to falls, in physical quality of life and in the average number of doctor visits and hospitalisations. However, these results showed statistically significant improvements only in self-care agency and the average number of doctor visits. As for self-care agency, surprisingly, the comparison group also experienced a significant improvement in this variable. Perhaps the assessment, which includes many questions about health and other health-related variables, raised awareness of health and care among the study participants —including comparison group participants—and thus the assessment itself encouraged them to perform self-care behaviours [49]. On the other hand, the average number of doctor visits was reduced by 33% and hospitalisation rate by 50% among participants who received the intervention in comparison with the comparison group who rose by 14% their hospitalisation rates. Several studies have established the relationship between frailty and hospitalisation, and between falls and emergency room visits and hospitalisation [4,10,50]. Therefore, a reduction of risk of falling and/or frailty could lead to a reduction of the use of health resources.

Finally, the cost-effectiveness analyses of the intervention conducted using the MAFEIP tool showed the potential for cost savings that our physical exercise programme provided in comparison to the usual care scenario, which was provided to the comparison group participants. Similar results have been found for other multicomponent programmes and in several systematic reviews and meta-analyses. The OTAGO Programme was cost-saving in terms of falls prevented in people older than 80 [51]. The reviews of Davis et al. [52] and Balzer et al. [53] showed that tailored multicomponent interventions for older adults suffering from risk of falling, that include balance and strength training, were cost-effective or cost-saving. Recently, the review of Apostolo et al. [22] examined the effectiveness and cost-effectiveness of interventions for frailty in older adults, concluding that physical exercise interventions showed to be generally effective for reducing frailty but only when conducted in groups. The economic estimation demonstrated that this type of intervention, compared to usual care, can provide better value for money.

The results of the present study should be interpreted in the context of potential limitations. First, the lack of randomisation in the distribution of participants in the intervention and comparison groups. Aware of such limitations, this study tested the homogeneity between groups by analysing the equivalence between the initial sample of subjects assigned to the intervention group and the comparison group. Another limitation of this research is the sample size, since this was reduced to 55 participants in the intervention group and 136 participants in the comparison group, which increased risk of type I error. However, it is important to note that the achieved sample size is considered appropriated in research [54] and the alpha value of the primary outcomes was ≤0.01, which reduces the probability of type I error. Nevertheless, the study results should be interpreted with caution and it is recommended to replicate the study with a deeper analysis of other relevant variables which could explain partially the positive effect of the intervention (motivation, socioeconomic variables, etc.).

## 5. Conclusions

In conclusion, the results of this study highlight the need to implement multicomponent physical exercise programmes to reduce the risk of falling and frailty, two of the most prevalent conditions which have a greater impact on older adults’ health. In this regard, our novel 9-month multicomponent physical exercise programme that offers a modified format of OTAGO programme including evidence-based improvements has demonstrated to be both potentially effective and less costly than usual care.

## Figures and Tables

**Table 1:** Intragroup and intergroup differences of risk of falling *.

Risk of Falling Differences	Intervention Group	Comparison Group	Intergroup Differences
**PRE *n* (%)**	50 (90.9%)	86 (63.2%)	X^2^ = 14.628; *p* = 0.000
**POST *n* (%)**	25 (45.4%)	87 (64.0%)	X^2^ = 5.536; *p* = 0.019
**% of change**	−45.5%	+0.8%	
**Intragroup differences**	McNemar; *p* = 0.000	McNemar; *p* = 1.000	
Effect size **	0.29	0.38	

* Intragroup differences were analysed using McNemar test and intergroup analyses using Chi-square test. ** Effect size effect was calculated using Cramer’s V (small effect = 0.2, medium effect = 0.5, large effect = 0.8).

**Table 2:** Intragroup and intergroup differences of frailty *.

Frailty Differences	Intervention Group	Comparison Group	Intergroup Differences
**PRE Mean ± SD**	6.20 ± 3.15	5.21 ± 2.24	U de Mann–Whitney = 3031.5; *p* = 0.039
**POST Mean ± SD**	4.27 ± 2.69	5.01 ± 2.26	U de Mann–Whitney = 3054.5; *p* = 0.046
**% of change**	−31%	−4%	
**Intragroup differences**	Wilcoxon z = −4.373; *p* = 0.000	Wilcoxon z = −1.142; *p* = 0.253	
Effect size **	0.59	0.09	

* Intragroup differences were analysed using Wilcoxon’s signed rank test and intergroup differences using Mann–Whitney U test. ** Effect size effect was calculated using r = Z/√N (r: effect size; Z: z value from Wilcoxon test; N: Observation number; small effect = 0.2, medium effect = 0.5, large effect = 0.8). Alternative to Cohen’s *d* when general assumptions of Cohen’s formula are violated (Rosenthal, 1994).

**Table 3:** Intragroup differences (intervention group only) of physical performance and body composition *.

Physical Performance and Body Composition Variables	PRE Mean ± SD	POST Mean ± SD	Pre-Post Difference	Effect Size **
**Physical Performance (SPPB)**				
**SPPB total**	7.71 ± 2.07	8.35 ± 2.15	*p* = 0.009	0.35
**Balance test**	3.18 ± 0.98	3.51 ± 0.84	*p* = 0.009	0.35
**Gait speed test**	2.44 ± 1.03	2.62 ± 1.01	*p* = 0.176	0.18
**Chair stand test**	2.09 ± 1.06	2.22 ± 1.17	*p* = 0.225	0.16
**Body composition (Tanita scale)**				
**Weight**	70.95 ± 14.02	70.39 ± 13.9	*p* = 0.152	0.07
**BMI**	30.96 ± 6.07	30.55 ± 5.95	*p* = 0.045	0.07
**Body fat (kg)**	27.61 ± 10.07	26.95 ± 9.87	*p* = 0.075	0.04
**Lean body mass (kg)**	43.34 ± 6.66	42.72 ± 8.52	*p* = 0.930	0.01
**Body water (kg)**	31.95 ± 4.78	31.82 ± 4.95	*p* = 0.868	0.02

* Intragroup differences were analysed using T-test and Wilcoxon’s signed rank test. ** Effect size was calculated using Cohen’s *d* for Weight, BMI and Body fat (small effect = 0.2, medium effect = 0.5, large effect = 0.8); the effect size for the other variables was calculated using r = Z/√N (r: effect size; Z: z value from Wilcoxon test; N: Observation number; small effect = 0.2, medium effect = 0.5, large effect = 0.8). SPPB: Short Physical Performance Battery.

**Table 4:** Intragroup differences of health-related variables *.

Health-Related Variables		Intervention Group	Comparison Group
**SF-12 Physical Health**	% of change	+7%	−9%
	Intragroup difference	*p* = 0.200	*p* = 0.001
	Effect size **	0.17	0.28
**SF-12 Mental Health**	% of change	−2%	−7%
	intragroup difference	*p* = 0.535	*p* = 0.003
	Effect size **	0.08	*0.25*
**Self-care agency**	% of change	+7%	+8%
	Intragroup difference	*p* = 0.001	*p* = 0.000
	Effect size **	0.48	0.42
**Limitation in ADLs**	% of change	−6%	+3%
	Intragroup difference	*p* = 0.011	*p* = 0.905
	Effect size **	0.35	0.01
**Falls self-efficacy**	% of change	−9%	+4%
	Intragroup difference	*p* = 0.098	*p* = 0.298
	Effect size **	0.22	0.09
**Average doctor visits**	% of change	−33%	−7%
	Intragroup difference	*p* = 0.000	*p* = 0.070
	Effect size **	0.48	0.15
**Hospitalisation**	% of change	−50%	+14%
	Intragroup difference	*p* = 0.508	*p* = 0.832
	Effect size **	0.08	0.18

* Intragroup differences were analysed using Wilcoxon’s signed rank test and McNemar test. ** Effect size were calculated using r = Z/√N (r: effect size; Z: z value from Wilcoxon test; N: Observation number; r can be interpreted as small effect = 0.1, medium effect = 0.3, large effect = 0.5). The effect size of hospitalisation was calculated using phi from McNemar test (Phi can be interpreted as small effect = 0.1, medium effect = 0.3, large effect = 0.5). ADLS: activities of daily living.

**Table 5:** Input data used to populate the Monitoring and Assessment Framework for the European Innovation Partnership on Active and Healthy Ageing (MAFEIP) model.

Variables Used in MAFEIP	Intervention Group	Comparison Group
**Costs**		
Recurring cost per patient/year (intervention) Healthcare cost—baseline Healthcare cost—deteriorated	104€ 1615.02€ 3130.96€	- 1630.22€ 9030.13€
Utility **		
Baseline state * Deteriorated state *	0.81 0.75	0.81 0.75

* Baseline state includes those participants who show an improvement in the risk of falls and/or frailty at the final evaluation and Deteriorated state includes those participants showing a worsening in the risk of falling and/or frailty or those that show no progression (after 9 months remain the same). ** Utility is the quality of life weight (utility of 1 would refer to the quality of life in perfect health and a utility of 0 would refer to no quality or dead).

**Table 6:** Incremental costs and health-related quality of life (HRQoL) effects (cost-effectiveness).

Incremental Cost and HRQoL * Effects	Effect Result
Incremental cost (healthcare)	−44,832.92
Incremental effects	0.513
Incremental cost-effectiveness ratio (healthcare)	Dominant

* HRQoL: health-related quality of life.

## Supplementary Materials

The following are available online at https://www.mdpi.com/1660-4601/16/12/2086/s1, S1: Multicomponent physical exercise programme.
