# Supplementary material for: Effectiveness and Estimation of Cost-Effectiveness of a Group-Based Multicomponent Physical Exercise Programme on Risk of Falling and Frailty in Community-Dwelling Older Adults
Source: Int J Environ Res Public Health. 2019 Jun 13;16(12):2086. doi: 10.3390/ijerph16122086 (PMC6617042; doi:10.3390/ijerph16122086)
Supplement: Supplementary file 1 [file ijerph-16-02086-s001.pdf]

MULTICOMPONENT PHYSICAL EXERCISE PROGRAMME

Exercise routine 1 (Back + Flexibility)

Exercise 1

- *Position*: Supine with arms extended out to each side.
- *Execution*: Draw the straightened arms up and overhead. Keep the arms and legs straight. Return to the starting position. Repeat.

Exercise 2

- *Position:* Quadruped with hands slightly in front of the shoulders.
- *Execution:* Pull the buttocks towards the heels and stretch the back. Repeat.

Exercise 3 *(from OTAGO)*

- *Position*: Standing up tall with the feet shoulder-width apart. Place the hands on the small of the back.
- *Execution*: Gently arch back. Repeat.

Exercise 4 *(from OTAGO)*

- *Position*: Standing up tall and place the hands on the hips. Do not move the hips.
- *Execution*: Turn as far as you can to the right, and then to the left. Repeat to each side.

Exercise 5

- *Position*: Sitting straight. Place both hands behind the head with fingers interlocked.
- *Execution*: Turn the torso to the right trying to touch the back of the chair with the hand. Repeat to each side.

Exercise 6

- *Position*: Standing up with the legs slightly bent. Place the feet shoulder-width apart and the torso leaning forward.
- *Execution*: Raise both arms and then extend them out to the side until they are parallel with the floor. Return to the starting position. Repeat.

Exercise routine 2 (Torso, Abs and Neck)

Exercise 1

- *Position*: Standing up tall. Place the feet shoulder-width apart. Raise the arms above the head.
- *Execution*: Contract the arms and chest muscles. Lower both arms and cross them in front of the chest. Return to the starting position. Repeat.

Exercise 2

- *Position*: Standing up tall. Place the feet shoulder-width apart. Make a fist with each of the hands. Place one on top of the other in front of your body.
- *Execution*: Push upward against the top fist while the top fist offers resistance. Repeat to each side.

Exercise 3

- *Position*: Standing up tall with extended arms straight down at the sides. Place the feet shoulder-width apart.
- *Execution*: Contract the chest, back and arm muscles. Repeat.

Exercise 4

- *Position*: Supine with arms extended out to each side like a T. Bend both knees to a 90-degree angle.
- *Execution*: Keep the knees together and bring them over to the right. Repeat to each side.

Exercise 5 *(from OTAGO)*

- *Position*: Sitting straight or standing up tall, facing forward.
- *Execution*: Slowly turn the head to the right, and then slowly turn the head to the left. Repeat to each side.

Exercise 6 *(from OTAGO)*

- *Position*: Sitting straight or standing up tall, facing forward.
- *Execution:* Place one hand on the chin. Guide the head straight back. Repeat.

Exercise routine 3 (Balance)

Exercise 1 *(from OTAGO)*

- *Position:* Sitting straight on a chair with armrests which is not too low.
- *Execution:* Place the feet behind the knees. Lean forward to the knees. Push off with both hands to stand up. Repeat.

Exercise 2 *(from OTAGO)*

- *Position:* Sitting straight on a chair with armrests which is not too low.
- *Execution:* Place the feet behind the knees. Lean forward to the knees. Push off with only one hand to stand up. Repeat.

Exercise 3 *(from OTAGO)*

- *Position:* Sitting straight on a chair with armrests which is not too low.
- *Execution:* Place the feet behind the knees. Lean forward to the knees. Stand up without using the hands. Repeat.

Exercise 4 *(from OTAGO)*

- *Position:* Standing up tall and hold on to a bench or table.
- *Execution:* Walk backwards 10 steps. Turn around and hold on with the other hand. Walk backwards 10 steps. Repeat.

Exercise 5 *(from OTAGO)*

- *Position:* Standing up tall and look ahead. Keep a bench or table close.
- *Execution:* Walk backwards 10 steps. Turn around. Walk backwards 10 steps. Repeat.

Exercise 6 *(from OTAGO)*

- *Position:* Standing up in front of stairs. Hold on to the hand-rail.
- *Execution:* Go up and down the stairs once.

Exercise routine 4 (Neck and Shoulders)

Exercise 1

- *Position*: Sitting straight or standing up tall. Keep the shoulders down.
- Execution: Bend the head forward (the chin moves towards the chest) and then, from the initial position bend the head backwards (the chin moves away from the chest). Repeat.

Exercise 2

- *Position:* Sitting straight or standing up tall. Keep the shoulders down.
- *Execution:* Bend the head to the right side (the right ear to the right shoulder). Repeat on other side.

Exercise 3

- *Position:* Sitting straight or standing up tall with extended arms straight down at the sides.
- *Execution:* Make arm circles forward and then back (start with small circles, then gradually larger circles).

Exercise 4

- *Position:* Standing up tall with extended arms straight down at the sides.
- *Execution:* Grab the right elbow with the left hand from behind the back. Move the right arm forward while the opposite arm offers resistance. Repeat on other side.

Exercise 5

- *Position:* Standing up tall. Bring the arms behind the back.
- *Execution:* Grab the right arm wrist with the left hand. Lift the right shoulder up toward the right ear while the opposite arm offers resistance. Repeat on other side.

Exercise 6

- *Position:* Standing up tall. Bend both arms at a 90-degree angle and place them at the waistline.

- *Execution:* Grab the right arm wrist with the left hand. Pull it while the opposite arm offers resistance. Repeat on other side.

Exercise routine 5 (Upper limb)

Exercise 1

- *Position:* Standing up tall. Bend the right arm at a 90-degree angle.
- *Execution:* Grab the right hand with the left hand. Push them together as hard as you can. Repeat on other side.

Exercise 2

- *Position:* Standing up tall with extended arms straight down at the sides.
- *Execution*: Bend the arms at the elbows and make fists. Squeeze the fists to the shoulders. Resist against the pressure as squeezing up. Repeat.

Exercise 3

- *Position:* Sitting straight. Place the right forearm on a bench or table with the palm facing upwards.
- *Execution:* Bend the arms at the elbows and make fists. Squeeze the fists to the shoulders. Resist against the pressure as squeezing up. Repeat.

Exercise 4

- *Position*: Sitting straight or standing up tall.
- *Execution:* Lift an arm forward. Bend the hands at the wrist. Repeat this on each arm.

Exercise 5

- *Position*: Sitting straight or standing up tall.
- *Execution*: Lift both arms forward. Rapid opening and closing of the hand.

Exercise 6

- *Position*: Sitting straight or standing up tall.
- *Execution*: Place both hands open and facing down. Bend the fingers one at a time towards the palm.

Exercise routine 6 (Balance)

Exercise 1 *(from OTAGO)*

- *Position*: Standing up tall beside a bench or table. Hold on to the bench or table and look ahead.
- *Execution*: Place on foot directly in front of the other foot, so the feet form a straight line. Hold this position for 10 seconds. Change position and place the foot behind directly in front. Hold this position for 10 seconds.

Exercise 2 *(from OTAGO)*

- *Position*: Standing up tall beside a bench or table and look ahead. Keep a bench or table close.

- *Execution:* Place on foot directly in front of the other foot, so the feet form a straight line. Hold this position for 10 seconds. Change position and place the foot behind directly in front. Hold this position for 10 seconds.

Exercise 3 *(from OTAGO)*

- *Position*: Standing up tall beside a bench or table. Hold on to the bench or table and look ahead.
- *Execution:* Stand on one leg for 10 seconds. Stand on the other leg for 10 seconds.

Exercise 4 *(from OTAGO)*

- *Position*: Standing up tall beside a bench or table and look ahead. Keep a bench or table close.
- *Execution:* Stand on one leg for 10 seconds. Stand on the other leg for 10 seconds.

Exercise 5 *(from OTAGO)*

- *Position*: Standing up tall beside a bench or table and look ahead. Keep a bench or table close.
- *Execution:* Stand on one leg for 30 seconds. Stand on the other leg for 30 seconds.

Exercise 6 *(from OTAGO)*

- *Position:* Standing up.
- *Execution:* Walk at regular pace. Turn in a clockwise direction. Walk back to the starting position. Turn in an anti-clockwise direction. The exercise in a figure of eight movement. Repeat.

Exercise routine 7 (Hips and Thighs)

Exercise 1 *(from OTAGO)*

- *Position*: Standing up tall beside a bench or table. Hold on to the bench or table. Keep the exercising leg straight and the foot straight forward.
- *Execution*: Lift the leg out to the side and return. Repeat this on each leg.

Exercise 2 *(from OTAGO)*

- *Position:* Standing up tall in front of a bench or table with both hands on it.
- *Execution*: Bend the knee, bringing the foot towards the bottom. Return to the starting position. Repeat this on each leg.

Exercise 3

- *Position:* Standing up tall. Keep a bench or table close.
- *Execution*: Lift one leg up to the side. Point the big toe toward the floor and start drawing semi-circles around the standing leg. Alternate from back to front, then front to back. Repeat this on each leg.

Exercise 4

- *Position*: Standing up with the legs slightly bent. Place one foot ahead of the other. Keep the body weight distributed on both sides.

- *Execution*: Contract the glutes while keeping the position. Repeat.

Exercise 5

- *Position*: Standing up tall and look ahead. Place the feet shoulder-width apart. Keep a bench or table close.
- *Execution:* Come up onto the toes. Squat down half way, bending the knees. Keep the heels elevated. Repeat.

Exercise 6 *(from OTAGO)*

- *Position*: Standing in front of a bench or table with both hands on it. Place the feet shoulder-width apart.
- Execution: Squat down half way, bending the knees. The knees go over the toes. When the heels start lifting, straighten up. Repeat.

Exercise routine 8 (Lower limb)

Exercise 1 *(from OTAGO)*

- *Position:* Standing up tall and look ahead. Place the feet shoulder-width apart. Keep a bench or table close.
- *Execution*: Squat down half way, bending the knees. The knees go over the toes. When the heels start lifting, straighten up. Repeat.

Exercise 2

- *Position*: Standing up tall. Place the feet together. Keep a bench or table close.
- *Execution*: Take a large step backward with one foot. Lower the back knee to a 90 degree angle so both knees are bent. Then press up to start position and repeat.

Exercise 3 *(from OTAGO)*

- *Position*: Sitting straight on a chair with the back well supported.
- *Execution*: Straighten the leg out. Lower the leg. Repeat this on each leg.

Exercise 4 *(from OTAGO)*

- *Position*: Sitting straight or standing up tall.
- *Execution*: Point the foot down then pull the foot back towards you. Repeat this for each foot.

Exercise 5 *(from OTAGO)*

- *Position*: Standing up in front of a bench or table. Hold on to the bench or table. Place the feet shoulder-width apart.
- Ex*ecution*: Come up onto the toes. Lower the heels to the ground. Repeat.

Exercise 6 *(from OTAGO)*

- *Position*: Standing up tall in front of a bench or table. Keep a bench or table close. Place the feet shoulder-width apart.
- *Execution*: Come up onto the toes. Lower the heels to the ground. Repeat.

Exercise routine 9 (Balance)

Exercise 1 *(from OTAGO)*

- *Position*: Standing up tall beside a bench or table. Hold on to it and look ahead.
- *Execution:* Come up onto the toes. Walk 10 steps on the toes. Lower the heels to the ground and turn around. Walk 10 steps on the toes. Repeat.

Exercise 2 *(from OTAGO)*

- *Position*: Standing up tall beside a bench or table and look ahead. Keep a bench or table close.
- *Execution:* Come up onto the toes. Walk 10 steps on the toes. Lower the heels to the ground and turn around. Walk 10 steps on the toes. Repeat.

Exercise 3 *(from OTAGO)*

- *Position*: Standing up tall with the hands on the hips.
- *Execution*: Take 10 side steps to the right and 10 side steps to the left. Repeat.

Exercise 4 *(from OTAGO)*

- *Position:* Standing up tall in front of a bench or table. Hold on to the bench or table.
- *Execution:* Place on foot in front of the other so they form a straight line. Place the foot behind directly in front. Repeat for 10 more steps. Turn around and repeat the exercise.

Exercise 5 *(from OTAGO)*

- *Position:* Standing up tall in front of a bench or table. Keep a bench or table close.
- *Execution:* Place on foot in front of the other so they form a straight line. Place the foot behind directly in front. Repeat for 10 more steps. Turn around and repeat the exercise.

Exercise 6 *(from OTAGO)*

- *Position*: Standing up tall in front of a bench or table. Keep a bench or table close.
- *Execution:* Place on foot directly behind the other foot. Place the foot in front directly behind. Repeat for 10 more steps. Turn around and repeat the exercise.

Exercise routine 10 (Calves and Feet + Balance)

Exercise 1 *(from OTAGO)*

- *Position:* Standing up tall in front of a bench or table. Hold on to the bench or table. Place the feet shoulder-width apart.
- *Execution:* Come back onto the heels, raising the front foot off the floor. Lower the feet to the ground. Repeat.

Exercise 2 *(from OTAGO)*

- *Position:* Standing up tall in front of a bench or table. Keep a bench or table close. Place the feet shoulder-width apart.
- *Execution:* Come back onto the heels, raising the front foot off the floor. Lower the feet to the ground. Repeat.

Exercise 3

- *Position*: Standing up tall. Place the feet shoulder-width apart and slightly bent knees.
- *Execution*: Shift your weight onto the outer edges of the feet while keeping the entire body upright. Repeat.

Exercise 4

- *Position:* Sitting straight. Rest the heels on the floor with the toes facing up.
- *Execution:* Bent downwards the toes of both feet towards the sole of the foot. Hold this position for few seconds. Repeat.

Exercise 5 *(from OTAGO)*

- *Position:* Standing up tall beside a bench or table. Hold on to it and look ahead.
- *Execution:* Come back onto the heels, raising the front foot off the floor. Walk 10 steps on the heels. Lower the feet to the ground and turn around. Walk 10 steps on the heels. Repeat.

Exercise 6 *(from OTAGO)*

- *Position:* Standing up tall beside a bench or table. Keep a bench or table close.
- *Execution:* Come back onto the heels, raising the front foot off the floor. Walk 10 steps on the heels. Lower the feet to the ground and turn around. Walk 10 steps on the heels. Repeat.
